# 3-(3-Methyl­phen­yl)-2-thioxo-1,3-thia­zolidin-4-one

**DOI:** 10.1107/S1600536809045863

**Published:** 2009-11-07

**Authors:** Durre Shahwar, M. Nawaz Tahir, Asma Yasmeen, Naeem Ahmad, Muhammad Akmal Khan

**Affiliations:** a Department of Chemistry, Government College University, Lahore, Pakistan; b Department of Physics, University of Sargodha, Sargodha, Pakistan

## Abstract

In the title compound, C_10_H_9_NOS_2_, the dihedral angle between the rhodanine (2-thioxo-1,3-thia­zolidin-4-one) and 3-methyl­phenyl rings is 83.30 (3)°. The H atoms of the methyl group are disordered over two set of sites with an occupancy ratio of 0.58 (3):0.42 (3). In the crystal, the mol­ecules inter­act by way of C—H⋯π and C=O⋯π inter­actions.

## Related literature

For related structures, see: Shahwar *et al.* (2009*a* ▶,*b* ▶,*c* ▶,*d* ▶,*e* ▶).
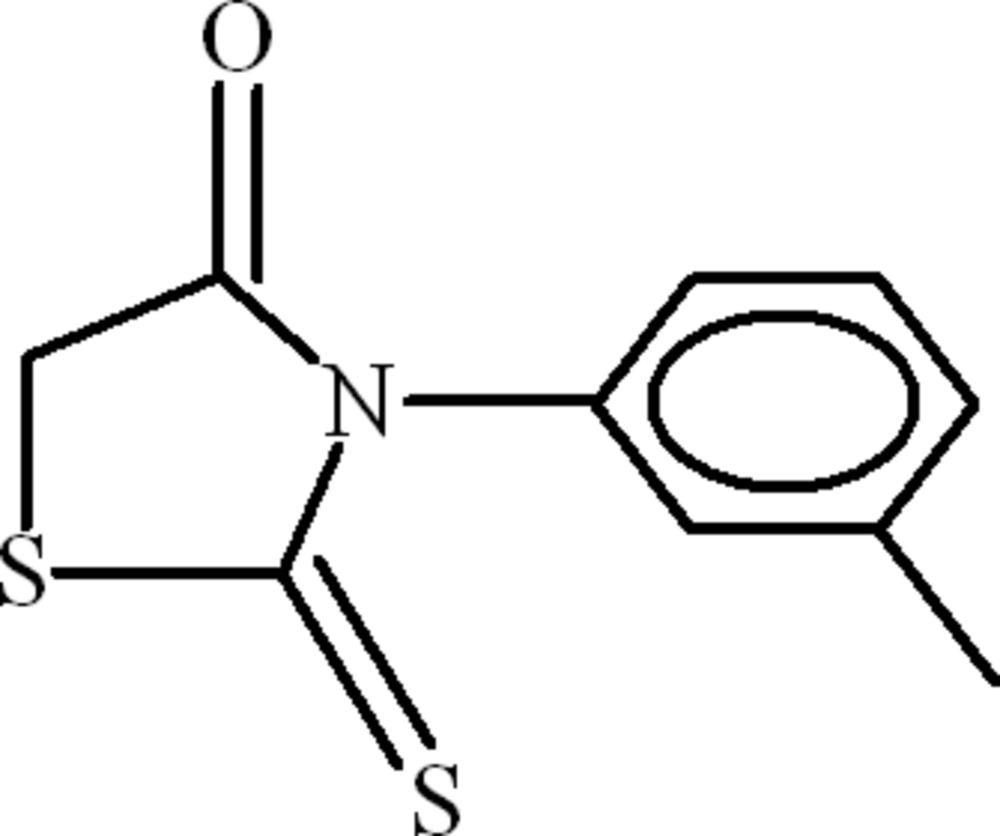


## Experimental

### 

#### Crystal data

C_10_H_9_NOS_2_
*M* *_r_* = 223.3
Monoclinic, 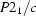

*a* = 8.0775 (3) Å
*b* = 6.4058 (2) Å
*c* = 21.4715 (7) Å
β = 106.068 (2)°
*V* = 1067.59 (6) Å^3^
*Z* = 4
Mo *K*α radiation
μ = 0.46 mm^−1^
*T* = 296 K
0.32 × 0.24 × 0.22 mm

#### Data collection

Bruker Kappa APEXII CCD diffractometer
Absorption correction: multi-scan (*SADABS*; Bruker, 2005 ▶) *T*_min_ = 0.849, *T*_max_ = 0.897
11841 measured reflections
2666 independent reflections
2116 reflections with *I* > 2σ(*I*)
*R*_int_ = 0.026

#### Refinement

*R*[*F* ^2^ > 2σ(*F* ^2^)] = 0.032
*wR*(*F* ^2^) = 0.087
*S* = 1.03
2666 reflections
129 parameters
H-atom parameters constrained
Δρ_max_ = 0.27 e Å^−3^
Δρ_min_ = −0.22 e Å^−3^

### 

Data collection: *APEX2* (Bruker, 2007 ▶); cell refinement: *SAINT* (Bruker, 2007 ▶); data reduction: *SAINT*; program(s) used to solve structure: *SHELXS97* (Sheldrick, 2008 ▶); program(s) used to refine structure: *SHELXL97* (Sheldrick, 2008 ▶); molecular graphics: *ORTEP-3 for Windows* (Farrugia, 1997 ▶) and *PLATON* (Spek, 2009 ▶); software used to prepare material for publication: *WinGX* (Farrugia, 1999 ▶) and *PLATON*.

## Supplementary Material

Crystal structure: contains datablocks global, I. DOI: 10.1107/S1600536809045863/hb5208sup1.cif

Structure factors: contains datablocks I. DOI: 10.1107/S1600536809045863/hb5208Isup2.hkl

Additional supplementary materials:  crystallographic information; 3D view; checkCIF report

## Figures and Tables

**Table 1:** Hydrogen-bond geometry (Å, °)

*D*—H⋯*A*	*D*—H	H⋯*A*	*D*⋯*A*	*D*—H⋯*A*
C8—H8*B*⋯*Cg*2^i^	0.97	2.59	3.5219 (17)	162
C7—O1⋯*Cg*1^i^	1.20 (1)	2.94 (1)	4.1070 (16)	164 (1)

Symmetry code: (i) 
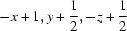
. *Cg*1 and *Cg*2 are the centroids of the S1/C8/C7/N1/C9 and C1—C6 rings, respectively.

**Fig. 1.**
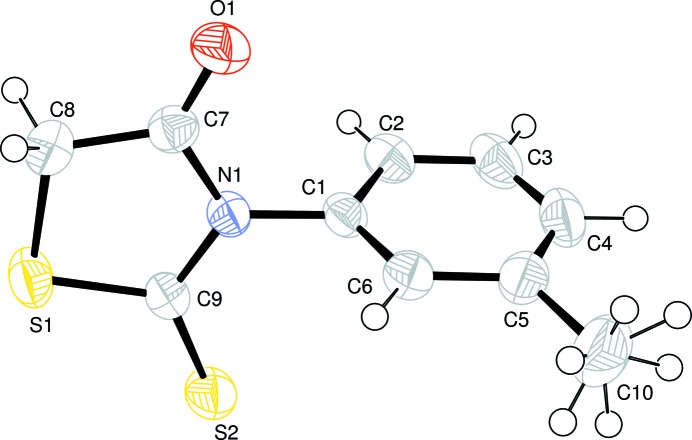
View of (I) with displacement ellipsoids drawn at the 50% probability level.

## supplementary crystallographic information

### Comment

We have reported the synthesis and crystal structures of various rhodanine derivatives such as (II) (5*Z*)-5-(2-Hydroxybenzylidene)-3-phenyl-2-thioxo-1,3-thiazolidin-4-one (Shahwar *et al.*, 2009*a*), (III) (5*E*)-5-(4-Hydroxy-3-methoxybenzylidene)-2-thioxo-1, 3-thiazolidin-4-one methanol monosolvate (Shahwar *et al.*, 2009*b*), (IV) (5*Z*)-5-(2-Hydroxybenzylidene)-2-thioxo-1,3-thiazolidin-4-one methanol hemisolvate (Shahwar *et al.*, 2009*c*), (V) 3-(2-Methylphenyl)-2-thioxo-1,3-thiazolidin-4-one (Shahwar *et al.*, 2009d) and (VI) 3-Cyclohexyl-2-thioxo-1,3-thiazolidin-4-one (Shahwar *et al.*, 2009e). The purpose of synthesis of differet rhodanine derivatives is to study the biological activities. The title compound (I, Fig. 1) is being reported in this context.

In (I), the 3-methylphenyl A (C1–C6/C10) and the rhodanine group B (N1/C7/C8/S1/C9/O1/S2) are planar with maximum r. m. s. deviations of 0.0068 and 0.0171 Å respectively, from their mean square planes. The dihedral angle between A/B is 83.30 (3)°. The H-atoms of the methyl moiety are disordered over two set of sites with occupancy ratio of 0.58 (3):0.42 (3) in the monomers. There exist C–H···π and C==O···π interactions (Table 1) which stabilize the molecules.

### Experimental

The title compound was prepared by a three step reaction procedure. In the first step *meta* toluidine aniline (10.7 g, 0.1 mol) and triethylamine (50.5 g, 0.5 mol) were stirred in ethanol (20 ml) followed by dropwise addition of CS_2_ (15.2 g, 0.2 mol) while keeping the flask in an ice bath. The precipitate obtained were filtered off and washed with diethyl ether.

In second step, a solution of sodium chloroacetate (11.6 g, 0.1 mol) and chloroacetic acid (18.9 g, 0.2 mol) was prepared in 50 ml distilled water. To this solution the precipitates obtained in first step were added gradually and stirred at 273 K. This mixture was stirred untill it turned light yellow.

In third step the yellow mixture was mixed in 140 ml hot (363–368 K) hydrochloric acid (6 N) and stirred for five minutes to obtain colorless crystalline precipitates. These precipitates were recrystalized in chloroform to get light yellow prisms of (I).

### Refinement

The H-atoms were positioned geometrically (C–H = 0.93–0.97 Å) and refined as riding with *U*_iso_(H) = 1.2*U*_eq_(C) or 1.5*U*_eq_(methyl C).

### Crystal data

**Table:**

C_10_H_9_NOS_2_	*F*(000) = 464
*M**_r_* = 223.3	*D*_x_ = 1.389 Mg m^−^^3^
Monoclinic, *P*2_1_/*c*	Mo *K*α radiation, λ = 0.71073 Å
Hall symbol: -P 2ybc	Cell parameters from 2666 reflections
*a* = 8.0775 (3) Å	θ = 2.6–28.3°
*b* = 6.4058 (2) Å	µ = 0.46 mm^−^^1^
*c* = 21.4715 (7) Å	*T* = 296 K
β = 106.068 (2)°	Prism, light yellow
*V* = 1067.59 (6) Å^3^	0.32 × 0.24 × 0.22 mm
*Z* = 4	

### Data collection

**Table:**

Bruker Kappa APEXII CCD diffractometer	2666 independent reflections
Radiation source: fine-focus sealed tube	2116 reflections with *I* > 2σ(*I*)
graphite	*R*_int_ = 0.026
Detector resolution: 7.40 pixels mm^-1^	θ_max_ = 28.3°, θ_min_ = 2.6°
ω scans	*h* = −10→10
Absorption correction: multi-scan (*SADABS*; Bruker, 2005)	*k* = −8→8
*T*_min_ = 0.849, *T*_max_ = 0.897	*l* = −28→28
11841 measured reflections	

### Refinement

**Table:**

Refinement on *F*^2^	Primary atom site location: structure-invariant direct methods
Least-squares matrix: full	Secondary atom site location: difference Fourier map
*R*[*F*^2^ > 2σ(*F*^2^)] = 0.032	Hydrogen site location: inferred from neighbouring sites
*wR*(*F*^2^) = 0.087	H-atom parameters constrained
*S* = 1.03	*w* = 1/[σ^2^(*F*_o_^2^) + (0.0399*P*)^2^ + 0.2635*P*] where *P* = (*F*_o_^2^ + 2*F*_c_^2^)/3
2666 reflections	(Δ/σ)_max_ < 0.001
129 parameters	Δρ_max_ = 0.27 e Å^−^^3^
0 restraints	Δρ_min_ = −0.22 e Å^−^^3^

### Special details

**Table:**

Geometry. Bond distances, angles *etc*. have been calculated using the rounded fractional coordinates. All su's are estimated from the variances of the (full) variance-covariance matrix. The cell e.s.d.'s are taken into account in the estimation of distances, angles and torsion angles
Refinement. Refinement of *F*^2^ against ALL reflections. The weighted *R*-factor *wR* and goodness of fit *S* are based on *F*^2^, conventional *R*-factors *R* are based on *F*, with *F* set to zero for negative *F*^2^. The threshold expression of *F*^2^ > σ(*F*^2^) is used only for calculating *R*-factors(gt) *etc*. and is not relevant to the choice of reflections for refinement. *R*-factors based on *F*^2^ are statistically about twice as large as those based on *F*, and *R*- factors based on ALL data will be even larger.

### Fractional atomic coordinates and isotropic or equivalent isotropic displacement parameters (Å^2^)

**Table:**

	*x*	*y*	*z*	*U*_iso_*/*U*_eq_	Occ. (<1)
S1	0.90452 (5)	0.42703 (7)	0.26996 (2)	0.0484 (1)	
S2	0.85641 (6)	0.38555 (7)	0.12935 (2)	0.0511 (2)	
O1	0.54228 (16)	0.04248 (19)	0.26950 (6)	0.0537 (4)	
N1	0.67528 (14)	0.18699 (18)	0.19889 (5)	0.0324 (3)	
C1	0.56186 (18)	0.0913 (2)	0.14230 (7)	0.0337 (4)	
C2	0.5937 (2)	−0.1103 (2)	0.12588 (8)	0.0419 (5)	
C3	0.4818 (2)	−0.1972 (3)	0.07148 (8)	0.0501 (6)	
C4	0.3436 (2)	−0.0858 (3)	0.03533 (8)	0.0518 (6)	
C5	0.3103 (2)	0.1156 (3)	0.05186 (7)	0.0461 (5)	
C6	0.42217 (18)	0.2036 (3)	0.10687 (7)	0.0391 (5)	
C7	0.65129 (19)	0.1553 (2)	0.26005 (7)	0.0363 (4)	
C8	0.77847 (19)	0.2774 (3)	0.31088 (7)	0.0410 (5)	
C9	0.80207 (17)	0.3243 (2)	0.19488 (7)	0.0342 (4)	
C10	0.1604 (2)	0.2403 (4)	0.01220 (10)	0.0730 (8)	
H2	0.68758	−0.18517	0.15069	0.0502*	
H3	0.50036	−0.33254	0.05925	0.0602*	
H4	0.27025	−0.14709	−0.00126	0.0621*	
H6	0.40257	0.33792	0.11964	0.0469*	
H8A	0.85188	0.18403	0.34219	0.0492*	
H8B	0.71912	0.36922	0.33352	0.0492*	
H10A	0.06610	0.23026	0.03116	0.1095*	0.58 (3)
H10B	0.12550	0.18649	−0.03121	0.1095*	0.58 (3)
H10C	0.19388	0.38382	0.01132	0.1095*	0.58 (3)
H10D	0.19424	0.31376	−0.02127	0.1095*	0.42 (3)
H10E	0.12470	0.33875	0.03963	0.1095*	0.42 (3)
H10F	0.06653	0.14807	−0.00708	0.1095*	0.42 (3)

### Atomic displacement parameters (Å^2^)

**Table:**

	*U*^11^	*U*^22^	*U*^33^	*U*^12^	*U*^13^	*U*^23^
S1	0.0426 (2)	0.0565 (3)	0.0445 (2)	−0.0166 (2)	0.0092 (2)	−0.0128 (2)
S2	0.0545 (3)	0.0578 (3)	0.0449 (2)	−0.0180 (2)	0.0202 (2)	−0.0016 (2)
O1	0.0636 (8)	0.0544 (7)	0.0476 (7)	−0.0196 (6)	0.0227 (6)	0.0006 (5)
N1	0.0328 (6)	0.0310 (5)	0.0331 (6)	−0.0024 (5)	0.0088 (5)	−0.0023 (5)
C1	0.0341 (7)	0.0342 (7)	0.0343 (7)	−0.0077 (6)	0.0120 (6)	−0.0027 (6)
C2	0.0433 (8)	0.0364 (8)	0.0482 (9)	−0.0029 (6)	0.0166 (7)	−0.0041 (7)
C3	0.0592 (10)	0.0442 (9)	0.0529 (10)	−0.0167 (8)	0.0253 (8)	−0.0169 (8)
C4	0.0521 (10)	0.0673 (11)	0.0375 (8)	−0.0261 (9)	0.0150 (7)	−0.0131 (8)
C5	0.0379 (8)	0.0627 (10)	0.0371 (8)	−0.0096 (7)	0.0094 (6)	0.0066 (7)
C6	0.0385 (8)	0.0399 (8)	0.0395 (8)	−0.0039 (6)	0.0119 (6)	0.0011 (6)
C7	0.0395 (8)	0.0343 (7)	0.0359 (7)	0.0028 (6)	0.0118 (6)	0.0018 (6)
C8	0.0388 (8)	0.0481 (9)	0.0351 (7)	0.0015 (7)	0.0087 (6)	−0.0026 (7)
C9	0.0314 (7)	0.0319 (7)	0.0389 (7)	−0.0006 (5)	0.0091 (6)	−0.0025 (6)
C10	0.0505 (11)	0.0965 (17)	0.0611 (12)	−0.0013 (11)	−0.0027 (9)	0.0161 (11)

### Geometric parameters (Å, °)

**Table:**

S1—C8	1.7952 (17)	C7—C8	1.496 (2)
S1—C9	1.7258 (15)	C2—H2	0.9300
S2—C9	1.6335 (15)	C3—H3	0.9300
O1—C7	1.199 (2)	C4—H4	0.9300
N1—C1	1.4406 (18)	C6—H6	0.9300
N1—C7	1.3943 (18)	C8—H8A	0.9700
N1—C9	1.3707 (18)	C8—H8B	0.9700
C1—C2	1.3814 (19)	C10—H10A	0.9600
C1—C6	1.376 (2)	C10—H10B	0.9600
C2—C3	1.381 (2)	C10—H10C	0.9600
C3—C4	1.371 (2)	C10—H10D	0.9600
C4—C5	1.384 (3)	C10—H10E	0.9600
C5—C6	1.392 (2)	C10—H10F	0.9600
C5—C10	1.503 (3)		
			
C8—S1—C9	93.63 (7)	C2—C3—H3	120.00
C1—N1—C7	120.71 (12)	C4—C3—H3	120.00
C1—N1—C9	122.13 (11)	C3—C4—H4	119.00
C7—N1—C9	116.98 (11)	C5—C4—H4	119.00
N1—C1—C2	119.55 (13)	C1—C6—H6	120.00
N1—C1—C6	118.46 (13)	C5—C6—H6	120.00
C2—C1—C6	121.98 (14)	S1—C8—H8A	110.00
C1—C2—C3	117.92 (15)	S1—C8—H8B	110.00
C2—C3—C4	120.60 (17)	C7—C8—H8A	110.00
C3—C4—C5	121.72 (16)	C7—C8—H8B	110.00
C4—C5—C6	117.93 (16)	H8A—C8—H8B	109.00
C4—C5—C10	122.24 (16)	C5—C10—H10A	109.00
C6—C5—C10	119.83 (17)	C5—C10—H10B	109.00
C1—C6—C5	119.84 (16)	C5—C10—H10C	109.00
O1—C7—N1	123.17 (14)	C5—C10—H10D	109.00
O1—C7—C8	125.44 (14)	C5—C10—H10E	109.00
N1—C7—C8	111.39 (12)	C5—C10—H10F	109.00
S1—C8—C7	106.86 (10)	H10A—C10—H10B	109.00
S1—C9—S2	122.64 (8)	H10A—C10—H10C	109.00
S1—C9—N1	111.07 (10)	H10B—C10—H10C	109.00
S2—C9—N1	126.29 (11)	H10D—C10—H10E	109.00
C1—C2—H2	121.00	H10D—C10—H10F	109.00
C3—C2—H2	121.00	H10E—C10—H10F	109.00
			
C9—S1—C8—C7	−2.42 (12)	C7—N1—C9—S2	179.24 (11)
C8—S1—C9—S2	−177.72 (10)	N1—C1—C2—C3	179.72 (14)
C8—S1—C9—N1	1.42 (11)	C6—C1—C2—C3	1.1 (2)
C7—N1—C1—C2	−85.14 (18)	N1—C1—C6—C5	179.89 (14)
C7—N1—C1—C6	93.56 (16)	C2—C1—C6—C5	−1.5 (2)
C9—N1—C1—C2	99.88 (17)	C1—C2—C3—C4	−0.2 (2)
C9—N1—C1—C6	−81.42 (18)	C2—C3—C4—C5	−0.4 (3)
C1—N1—C7—O1	3.2 (2)	C3—C4—C5—C6	0.0 (3)
C1—N1—C7—C8	−177.29 (12)	C3—C4—C5—C10	179.27 (17)
C9—N1—C7—O1	178.41 (14)	C4—C5—C6—C1	0.9 (2)
C9—N1—C7—C8	−2.06 (18)	C10—C5—C6—C1	−178.41 (15)
C1—N1—C9—S1	175.30 (10)	O1—C7—C8—S1	−177.61 (13)
C1—N1—C9—S2	−5.60 (19)	N1—C7—C8—S1	2.88 (16)
C7—N1—C9—S1	0.14 (16)		

### Hydrogen-bond geometry (Å, °)

**Table:**

*D*—H···*A*	*D*—H	H···*A*	*D*···*A*	*D*—H···*A*
C8—H8B···Cg2^i^	0.97	2.59	3.5219 (17)	162
C7—O1···Cg1^i^	1.199 (2)	2.9413 (14)	4.1070 (16)	163.94 (11)

Symmetry codes: (i) −*x*+1, *y*+1/2, −*z*+1/2.
